# Flatfoot over the centuries: the background of current conservative and operative treatments

**DOI:** 10.1007/s00264-023-05837-3

**Published:** 2023-05-24

**Authors:** Carlo Biz, Mariachiara Cerchiaro, Fabiana Mori, Alessandro Rossin, Mattia Ponticiello, Alberto Crimì, Pietro Ruggieri

**Affiliations:** Orthopedics and Orthopedic Oncology, Department of Surgery, Oncology and Gastroenterology DiSCOG, University of Padova, Via Giustiniani 2, 35128 Padua, Italy

**Keywords:** Flatfoot, Pes planus, Insoles, Foot deformities, Arthrodesis, Arthrorisis, Osteotomies, Tendon lengthening, Tendon transfer

## Abstract

**Purpose:**

Although flatfoot is a widespread human condition, historical medical texts and ancient illustrations on this deformity are extremely rare. Nowadays, doubts regarding its management remain unsolved. This historical review aims to identify the presence of pes planus since the prehistoric era and examine the treatments proposed over the centuries up to the present.

**Method:**

For this propose, we performed an extensive electronic search of the relevant literature, complemented by a manual search of additional sources from archaeological to artistic, literary, historical, and scientific accounts, describing flatfoot and its treatment in different eras.

**Results:**

Flatfoot accompanied the evolutionary timeline of human species: from Lucy *Australopithecus* to *Homo Sapiens*. It was described among various diseases suffered by *Tutankhamun *(1343–1324 B.C.), while the first anatomical description dates to *Emperor Trajan *(53–117 A.D.) and the medical studies of *Galen *(129–201 A.D.). It was also represented in the anatomical drawings of *Leonardo da Vinci *(1452–1519) and *Girolamo Fabrici d'Acquapendente *(1533–1619). Historically, the conservative treatment by insoles was the only one proposed until the nineteenth century. Since then, the most popular surgical procedures performed for correction have been osteotomies, arthrodesis, arthrorisis, and tendon lengthening and transfer.

**Conclusion:**

During the centuries, conservative therapeutic strategies have not radically changed in their substance, while operative ones have become the protagonists during the twentieth century up to the present. Nevertheless, after more than 2000 years of history, there is no consensus regarding the best indication for the flatfoot and if it really needs to be treated.

## Introduction

*Flatfoot* or *pes planus* is a condition that has been defined in various ways over time. Most commonly, it has been simply described as a foot with the medial margin of its plantar aspect in complete contact with the ground. The aetiology of its most common forms has been ascribed to an atavism in the development of human being [1]. Krogman (Oak Park, Illinos 1903 - Litiz, Pennsylvania 1987) interestingly described the pes planus as a “scar” of human evolution, observing that “[…] Our fallen arch trouble, our bunions, our calluses, and our foot miseries generally hark back to the fact that our feet are not yet healed by adaptation and evolutionary selection into really efficient units” [1]. Other authors, Helfet and Lee, described the pes planus as a condition in which the medial longitudinal arch is lower than “normal” on weight-bearing [2]. Instead, the term “pes planovalgus” was introduced to describe a more complex form, where both the arch is flat in the sagittal plan and the heel is in a pronounced valgus position everted in the frontal plane [2].

Currently, normal adult feet are described as having a characteristic morphology with the presence of two arches that allow for the correct discharge of forces during standing, walking, running, and jumping. Essential for the stability of the plantar arches are the dynamic musculo-tendinous structures: the tibialis posterior tendon, tendons of the flexor hallucis longus and flexor digitorum longus muscles, the peroneus longus and brevis muscle, the plantar calcaneonavicular and the deltoid ligaments, and the plantar aponeurosis [3]. Although most forms of pes planus are a widespread condition across time periods, unlike clubfoot, historical medical texts and ancient illustrations on its evolution throughout history to contemporary scientific literature are extremely rare [4]. The goal of this historical review is to try to fill this gap.

## The evolution of feet: from quadrupeds to bipeds

The foot is described as a part of the human body with an extraordinary number of anatomical evolutionary adaptations due to bipedalism and upright position. This is related to the disappearance of the opposability of the hallux in hominins and the consequent loss of the foot function as a grasping element [5].

In 1997, the orthopaedist *Albert Isidro Llorens* reported that in the primate evolution context, bipedalism is an anatomical adaptation to survive geo-climatic changes [6]. In 1809, Lamarck in his *Philosophie zoologique* [7] wrote that quadrupedal apes inhabited trees and only changed into pedals when they became fully terrestrial, probably because trees disappeared. In Darwin’s *Descent of Man* [8], it is argued that bipedalism emerged when an ancient primate became less arboreal conferring selective advantage because it freed hands from locomotion so they could be used to hold weapons and dominate other animals. Bipedal specialisation was found in ancient hominin such as *Ardipithecus ramidus*, lived about 4.4 million years (Myr) ago, and *Austrolopithecus anamensis*, lived about 4.2 Myr ago.

**Fig. 1:**
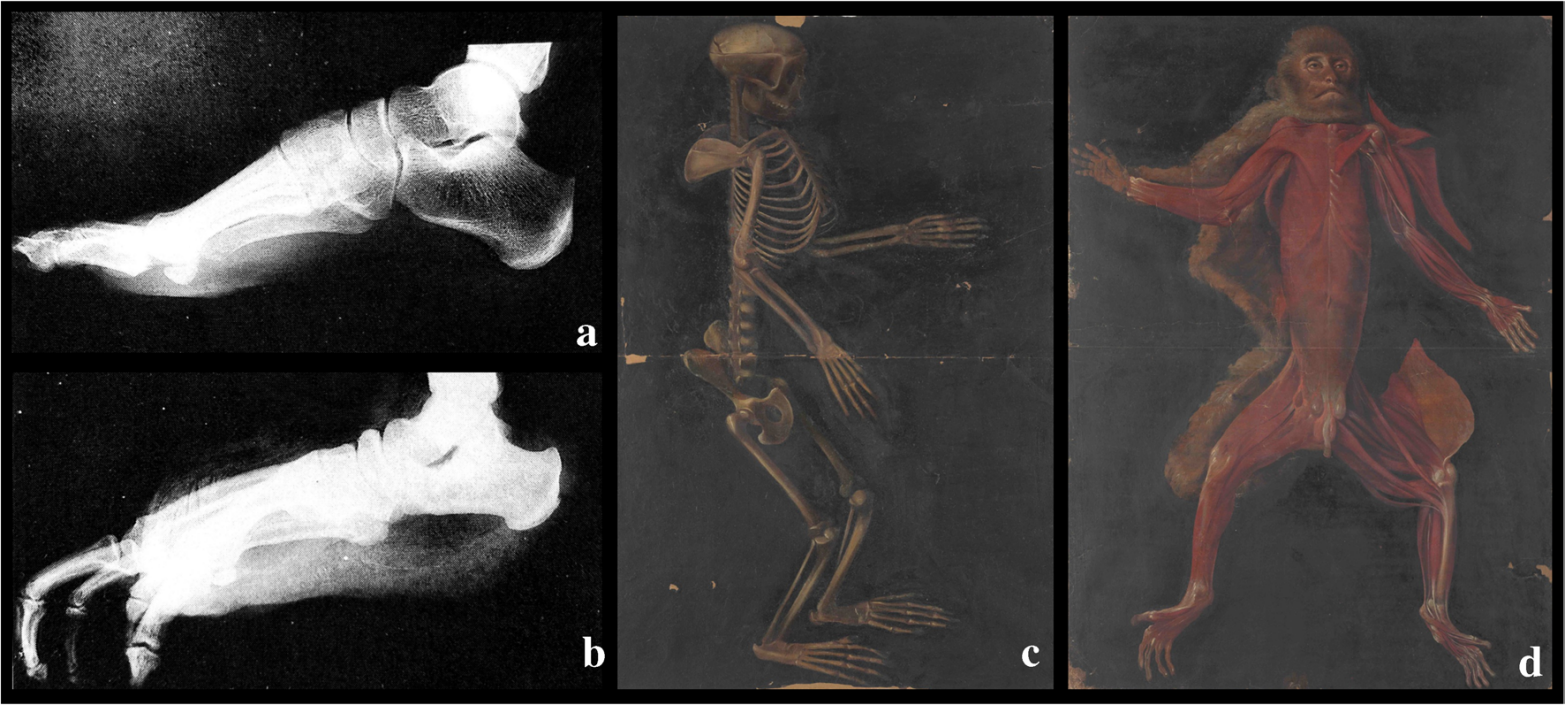
The evolution of foot in primates: **a** Lateral, not weightbearing, radiographic images of a human foot and **b** chimpanzee foot, modified from Donald Johanson, Blake Edgar, *From Lucy to Language*, s.l., Cassell & Co London, 2001; **c** and **d** anatomical images of the transverse arch in the monkey, from *Theatrum totius animalis fabricae - De anatomia animalium, Rari 113*, by Girolamo Fabrici d'Acquapendente, Marciana National Library in Venice

In 2016, Hatala and colleagues supposed that Pliocene hominin gained bipedal posture, conclusion based on studies conducted on the footprints discovered in 1977 at Laetoli, Tanzania. These trackways date back to around 3.66 Myr and are widely considered to have been made by *Australopithecus afarensis* [9]. In modern humans, foot bones are strong structures, able to hold the weight of the body to allow a walking bipedal gait [10]. It is recognised that all primates, such as chimpanzees, possess a transverse arch, but only humans have a longitudinal arch, making non-human primates anatomically and functionally flat-footed [11] (Fig. 1).

**Fig. 2:**
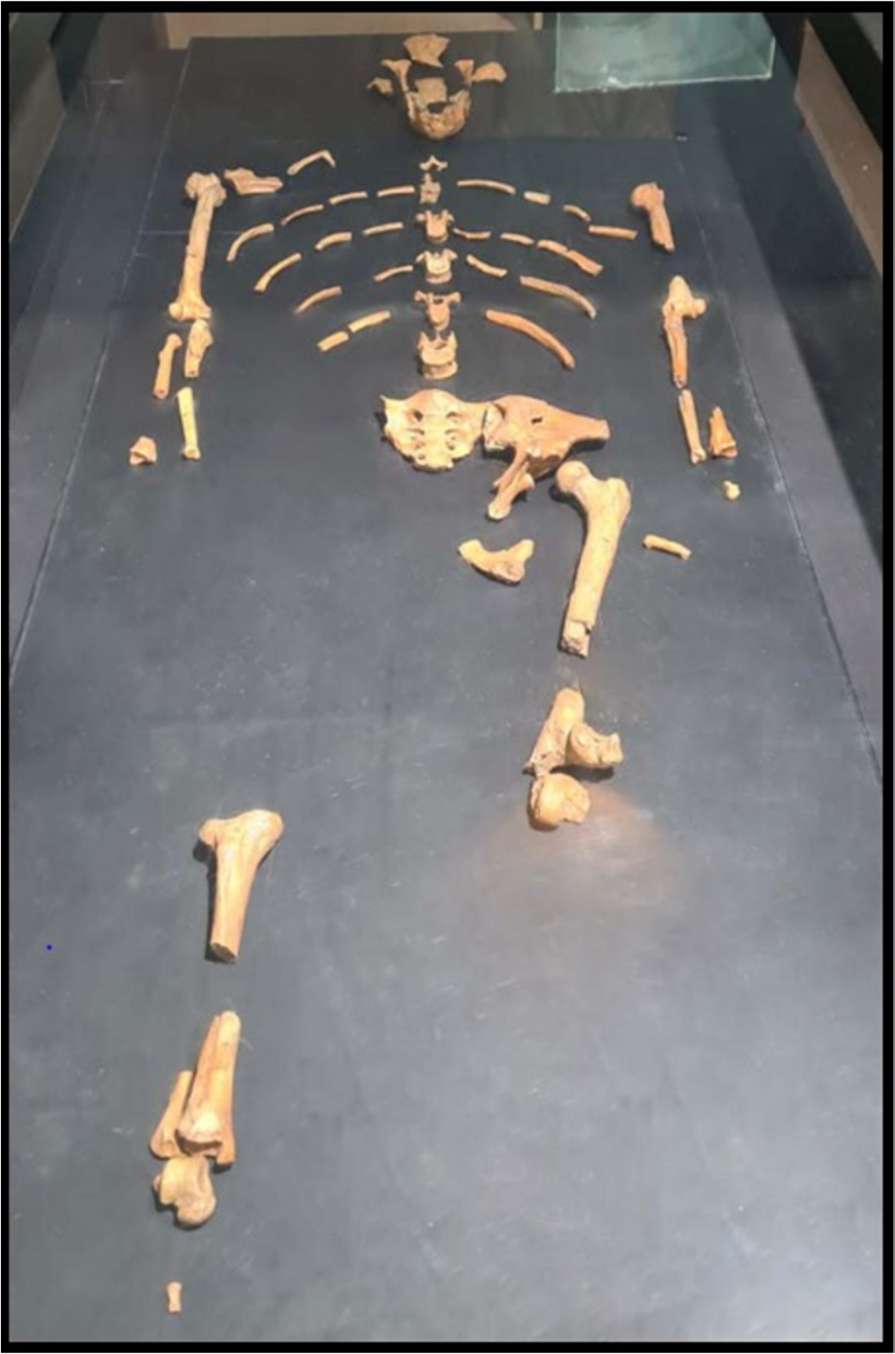
“Lucy” partial skeleton found at the sites of Hadar in Ethiopia in 1974 by Donald Johanson. The skeleton is dated to about 3.2 million years ago, and it has a small skull, 1.07 m high, and probably weighed between 29 and 45 kg. This original fossil of Lucy is kept at the National Museum of Ethiopia in Addis-Ababa

In the 1970s, mid-Pliocene hominin fossils were found at the site of Hadar in Ethiopia. The samples constituted the first substantial evidence for hominins older than three million years and were notable for some remarkable discoveries, such as the “Lucy” partial skeleton [12]. She was firstly described in 1978 by his discoverer D. Johanson in the manuscript “Kirtlandia” as a species of *Australopithecus* distinguished by a dentition with large upper central incisors with flexed roots, and asymmetric canines; a characteristic mandibular shape with an ascending ramus broad and post-canine teeth aligned in straight rows; a cranium featured by palate shallow, especially anteriorly; and dental arcade long, narrow, and straight-sided. Moreover, he described a high level of robusticity in all skeletal elements and pelvic region and lower limbs with bipedal locomotion adaptation [13]. By the studies conducted on bones of the famous 3.0–3.7-Myr-old hominin *Australopithecus afarensis* (this name derives from the Afar depression in Ethiopia where the largest portion of the paratype series was recovered [13]), it was found that “Lucy” had a flatfoot, although two other tibiae from *Hadar* suggest the presence of a rearfoot arch in the same species [14] (Fig. 2).

However, hominids, known as Archaic Primates, Prosimians, and Plesiadapiforms from the Paleocene and Eocene, show a traverse arch as reported by Conroy in his paper [15]. Considering the research in the fore/midfoot, the Kromdraai talar remains (*Paranthropus robustus*) exhibit similar features to those of Olduvai Hominid 8 (OH8), with its horizontal angle being more in line with apes (Gorilla gorilla, Pan troglodytes) than humans (continental Europeans, Bushmen, Anglo-Saxon/Romano-British). On the other hand, other studies proposed that the locomotion of *Australopithecus sediba* shows a unique form of bipedalism and a minor degree of arboreality, derived from its primitive and hominin foot. Its feet show a cuboid facet at a similar angle in line with humans, suggesting foot arching and a smooth calcaneal tuber surface.

Moreover, research conducted on the remains of the *Homo floresiensis* concluded that its feet show primitive features for the genus *Homo*, such as long lateral toes, a short first metatarsal, re-evolving short hind limbs, and flatfoot, among others [16].

The available evidence implies that there were several stages in the evolution of the arch of the human foot. First, apes such as chimpanzees and presumably the last common ancestor of apes and hominins lacked both a longitudinal and transverse arch. By 3.4 million years ago, though possibly earlier, a human-like transverse arch had evolved in *A. afarensis*, which however lacked a fully developed longitudinal arch [17]. Finally, in the genus *Homo*, we see a full longitudinal and transverse arch, enabling both effective walking and running [11]*.* The evolution of medial longitudinal arch can be dated to the attachment of the spring ligament in the talus found in individual OH 8 of *Homo habilis* (2.4–1.4 Myr) [18].

## Since the Neanderthals to the first pair of shoes

The proportions and bone morphology of the Neanderthal foot are similar to those of *Homo sapiens*, with the exception of the talus. Neanderthals external talus morphology reflects the various adaptations associated with their presumably hunter-gatherer, shoeless lifestyle unlike *Homo sapiens* who began wearing footwear. Studies suggest that higher human body mass of Neanderthals and/or higher mechanical stress led to their habitually pronated foot posture [19]. Neanderthals apparently did not wear hard-soled shoes, but covered their feet with skins to keep warm. Archaeological evidence suggests that footwear was in use from at least the Middle Upper Paleolithic (Gravettian) in parts of Europe. A comparative biomechanical analysis of the proximal phalanges of the foot of Western Middle Paleolithic and Middle Upper Paleolithic Eurasian humans indicates that supportive footwear was rare in the Middle Paleolithic, but became frequent in the Middle Upper Paleolithic [20]. According to anthropologist Erik Trinkaus, the smallest toes on our extremities weakened during that time, an anatomical change that the expert attributes to the invention of rudimentary shoes. These, in fact, reduced the need for strong and flexible fingers to grip the ground and balance the step. Ancestors who lived in cold climates may have started covering their feet to insulate them from the cold as early as 500,000 years ago. Until now, it has been difficult for archaeologists to determine exactly when humans stopped walking barefoot, not least because the plant and animal materials used for prehistoric shoes were highly perishable. Notably, Neanderthals and early *Homo sapiens* of the Middle Paleolithic (100,000 to 40,000 years ago) still had much stronger and more agile fingers than the Upper Paleolithic peoples, who inhabited the earth 26,000 years ago [20].

## The myth of Tutankhamun

It is necessary to fast forward to the Egyptian era to find the most famous ancient case of flatfoot: the Theban boy-king *Tutankhamun*. He was a scion of the 18th dynasty of the New Kingdom, the most powerful period of ancient Egypt (circa 1550–1070 B.C.). Nowadays, *Tutankhamun* is the most worldwide known pharaoh, although his tenure was brief because he died in the ninth year of his reign, about 1324 B.C., at 19 years of age [21].

**Fig. 3:**
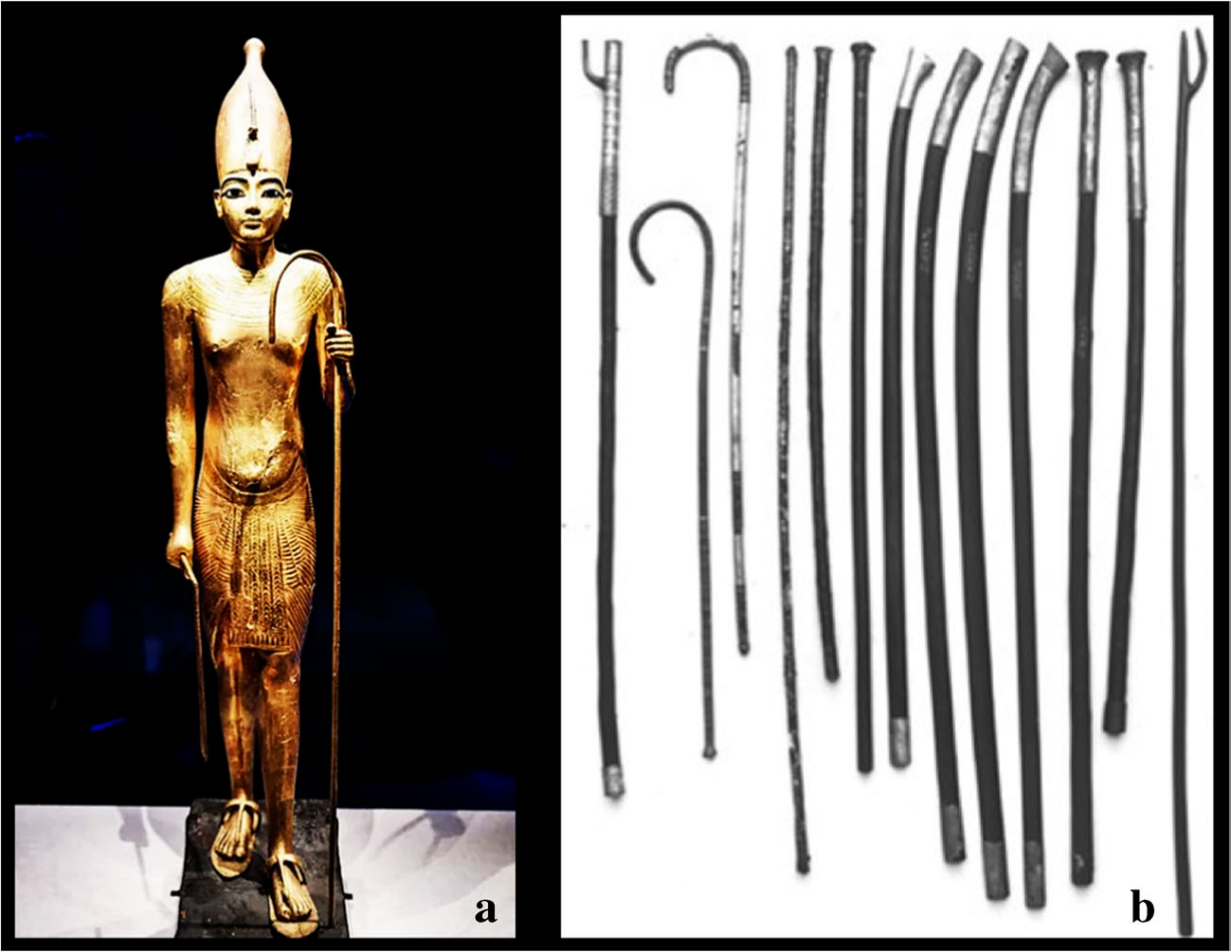
Pharaoh Tutankhamun (1343–1324 B.C.): **a** wooden statue of the Pharaoh Tutankhamun and his stick, Saatchi Gallery (London, UK), suggesting that the pharaoh may have used it to support himself; **b** walking sticks found in his tomb, Burton photograph, kept in the Griffith Institute Archive, University of Oxford

The weak pharaoh suffered from foot pathologies as supported by the King’s grave goods present in tomb KV62, discovered in the valley of kings by the British archaeologist and Egyptologist Howard Carter (London 1874–London 1939) [22] in November 1922. Although the pharaoh led a physically active life and had a normal body height for adolescence (1.67 m) [23], about 130 walking sticks were found in his tomb. Further, several images show the King with a long stick or a long sceptre; some suggest that the pharaoh may have used them to support himself (Fig. 3).

Tutankhamun’s father, *Akhenaton *(Tebe 1375 B.C. circa–Akhetaton 1334 B.C. circa), with the same A + blood group [24], and his mother (perhaps *Kiya* or *Nefertiti*), were probably brother and sister, passing on genetic defects to their children. All findings collected between 1922 and 2020, including computed tomography (CT) and genetic disease analysis, showed that the King suffered from several diseases in his lower limbs: pes planus, painful Kohler’s disease and oligodactyly of the right foot, and club foot and bone necrosis of metatarsal bones II–III of the left one [24].

In 2010, the Egyptologist Zahi Abass Hawass reported the detailed examination of the King’s right foot, proving the presence of a low longitudinal arch with Rocher angle of 132° (normal value, 126°) [23]. Hawass demonstrated that Tutankhamun had juvenile aseptic bone necrosis of the left 2nd and 3rd metatarsals, also involving the 2nd metatarso-phalangeal joint, due to an ulcerative osteoarthritis, probably the final outcome of osteochondral lesions. The malformed 2nd toe of the left foot together with the congenital equinovarus deformity of the left foot transferred the additional joint load to the right foot, probably causing the flattening of the foot arch [23].

In 1971, Helck [25] proposed that the most probable cause of death of *King Tutankhamun*, as well as that of his possible relative *Smenkhare*, could be connected to a sort of plague [26], as advocated by other authors [27–29]*.* Although the latest evidence indicates that the young king suffered from malaria [23], these authors believe that drepanocytosis and malaria may not have been the only cause of death, rather a contributing factor, combined with an infection after a complex fracture of the distal right femur (the fracture type corresponds to 33C3 of the AO classification) [23, 24, 30]. In relation to whether malaria was the primary cause of death, Timmann and Meyer suggested that these findings seem to be related to Gaucher disease rather than to anaemia falciparum [31]. For these reasons, the actual cause of death remains debated [32, 33].

## The most ancient image and the first description of flatfoot

Another interesting finding regarding flatfoot dates to the first century A.D. during the reign of the emperor *Trajan *(Italica 53 A.D.–Selinunte 117 A.D.) in the town of Ephesus, where an engraved pictogram footprint of a flatfoot on the Marble Road was found [4]. This road led to the great theatre and to the Celsus Library, as a portion of the sacred way that led to the Temple of Artemis [34].

To our knowledge, this engraved pictogram is probably among the oldest representations of a flatfoot in history [4]. It consists of three symbols: the foot, a female head with a bust, and a pierced heart opposite the female figure, symbolising the woman waiting in a brothel and her eagerness for love. It was thus believed to represent an advertisement for a brothel, and it is a realistic illustration of a flat left foot. By the studies of *Wokaunn*, *Ferenčić*, and* Mikolaučić* in 2013, the flatfoot diagnosis has been confirmed by arch index calculation [4].

Probably the foot belongs to an adult man with a foot that was size 39/40 by current standards (EU sizing). He could have been a worker deployed to road construction or perhaps an artist expressing his inspiration or a seafarer who came from the port in search of some pleasure or simply a local passer-by.

**Fig. 4:**
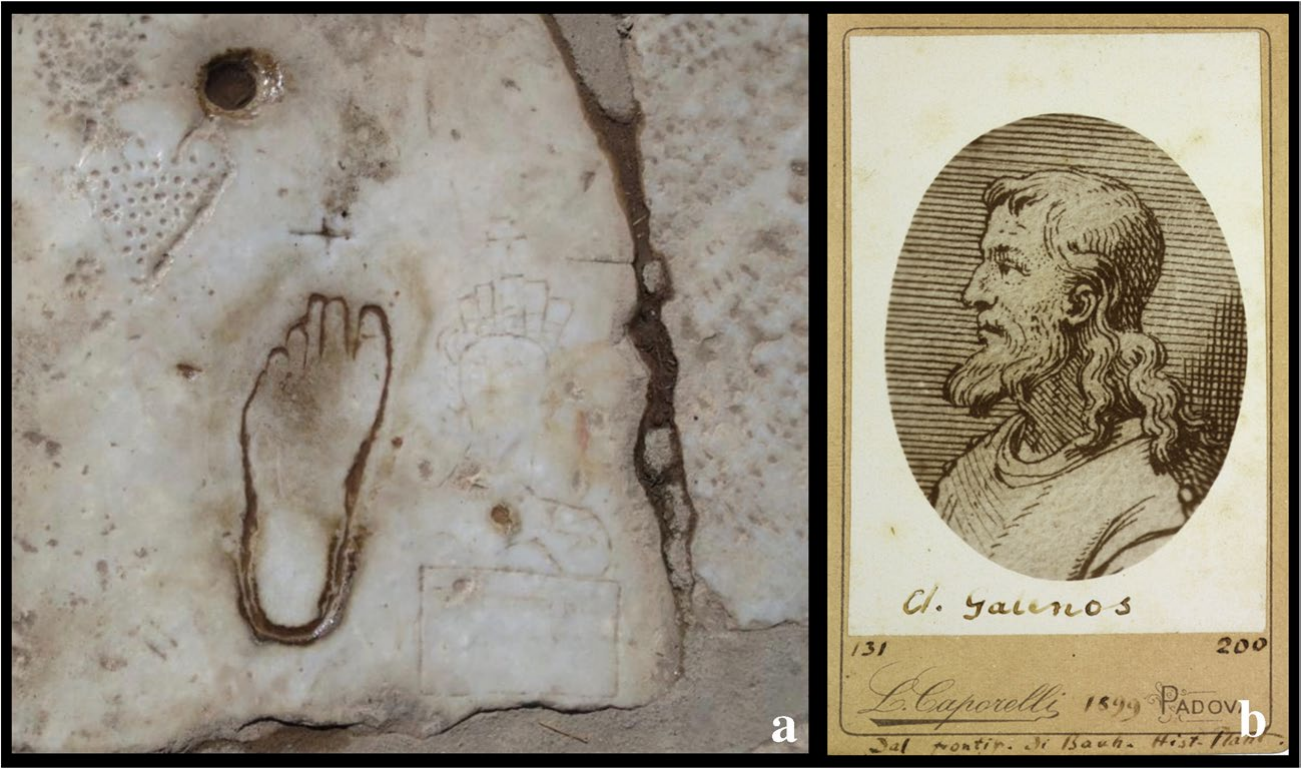
Galen and Ephesus: **a** the imprint of the left foot in the ancient city of Ephesus; **b** portrait of Galen from *The Botanical Garden Library, University of Padova*

In the same era, *Galen *(Pergamum, 129 AD–Rome 201 AD), the most famous Greek physician of antiquity after Hippocrates, was the first author to describe the pes planus as a deviation from normal foot anatomy, characterising the patients as “λειοποδες” (liopothes), which means people with smooth feet [35]. This can be considered the first historical definition of the pes planus in the second century A.D. Galen deals with the bony anatomy of the foot in his book *On Bones for Beginners* in chapter 24 dedicated to the tarsus and in chapter 25 describing the metatarsus (pethion) and the toes (Fig. 4).

## The anatomical studies of Leonardo Da Vinci and Girolamo Fabrici d’Acquapendente

No significant advances or developments in medical practices occurred after the fall of the Roman Empire until the Renaissance, a period of progress in European medical knowledge with renewed interest in the ideas of the ancient Greek and Roman civilisations.

**Fig. 5:**
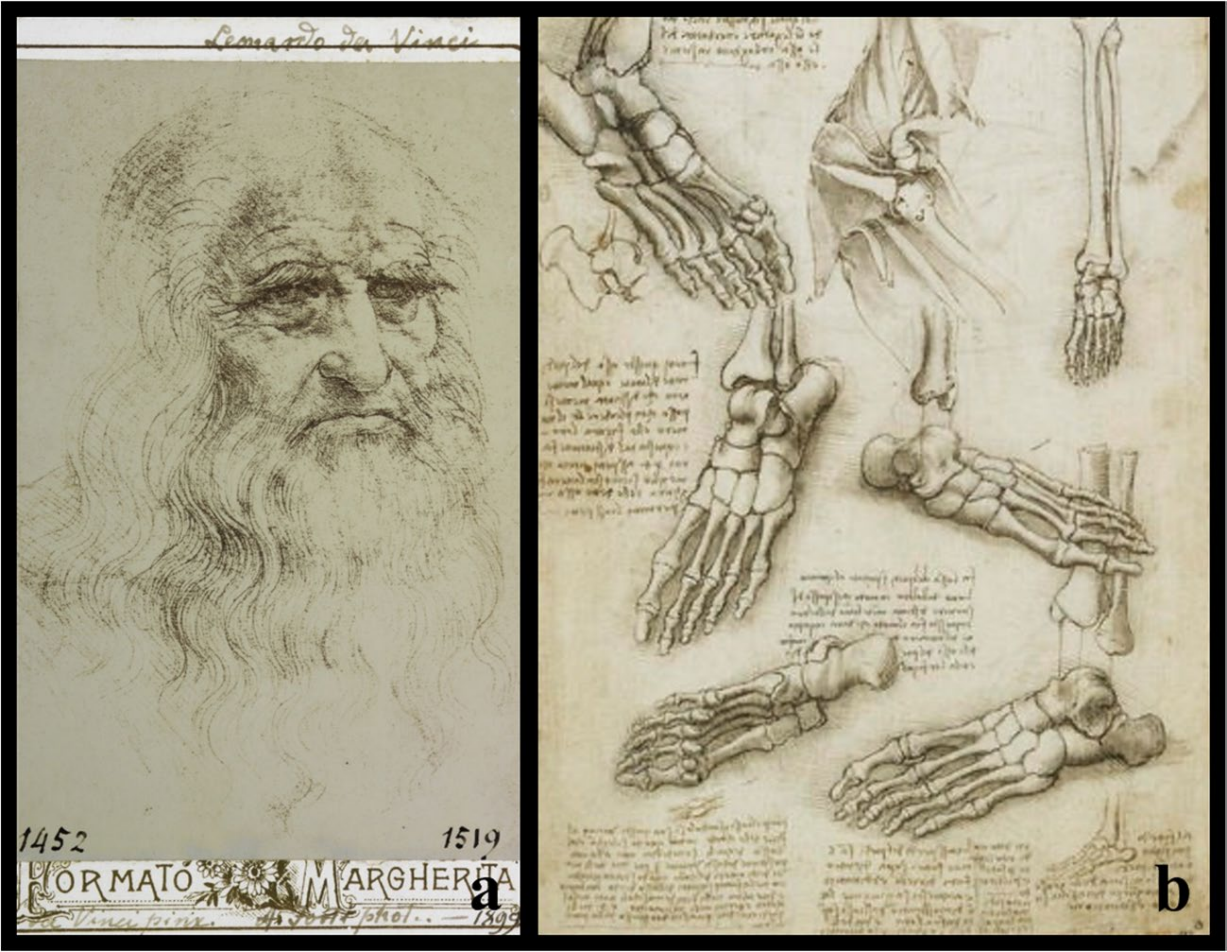
Leonardo Da Vinci (1452-1519): **a** portrait of the Italian artist and anatomist, from *The Botanical Garden Library*, University of Padova; **b** drawing of the foot bones from *Anatomic Manuscript A, from The Royal British Library*

*Leonardo da Vinci *(Anchiano 1452–Amboise 1519) became the unassailable icon of this era with his contributions in the fields of science, anatomy, and technology [36]. Evidenced mostly in his over 13,000 pages of drawings and notebooks, his interests in anatomy and mechanics were well documented, including an understanding of how he believed the foot and ankle worked, stating: “The human foot is a masterpiece of engineering and a work of art”. Most of these drawings were created between 1510 and 1511 and gathered in *Anatomic Manuscripts A*, *B*, and *C*, now preserved in Windsor Castle as part of the Royal Collection [37]. He performed about 30 cadaver dissections at Santa Maria Nuova Hospital in Florence and the Santo Spirito Hospital in Rome. In *Anatomic Manuscript A*, some drawings of dissected and prepared specimens with soft tissues stripped off of a pes planus deformity are shown. Leonardo intended to publish this material as an illustrated treatise, but at his death on 2 May 1519, the drawings remained among his private papers and were bequeathed to his young disciple, *Francesco Melzi* (Milano, 1491–Vaprio d'Adda, 1570) [38] (Fig. 5).

In the same era, *Girolamo Fabrici d’Acquapendente *(Acquapendente 1533–Padova 1619), an Italian anatomist, surgeon, and physiologist from Padua University, considered a precursor of modern orthopaedics, invented an external corrective device for the treatment of congenital and acquired deformities, the Oplomochlion. In 1594, he founded the first permanent anatomical theatre, still preserved in the Palazzo del Bo in Padua, in which he could study and teach anatomy based on cadaveric dissections.

**Fig. 6:**
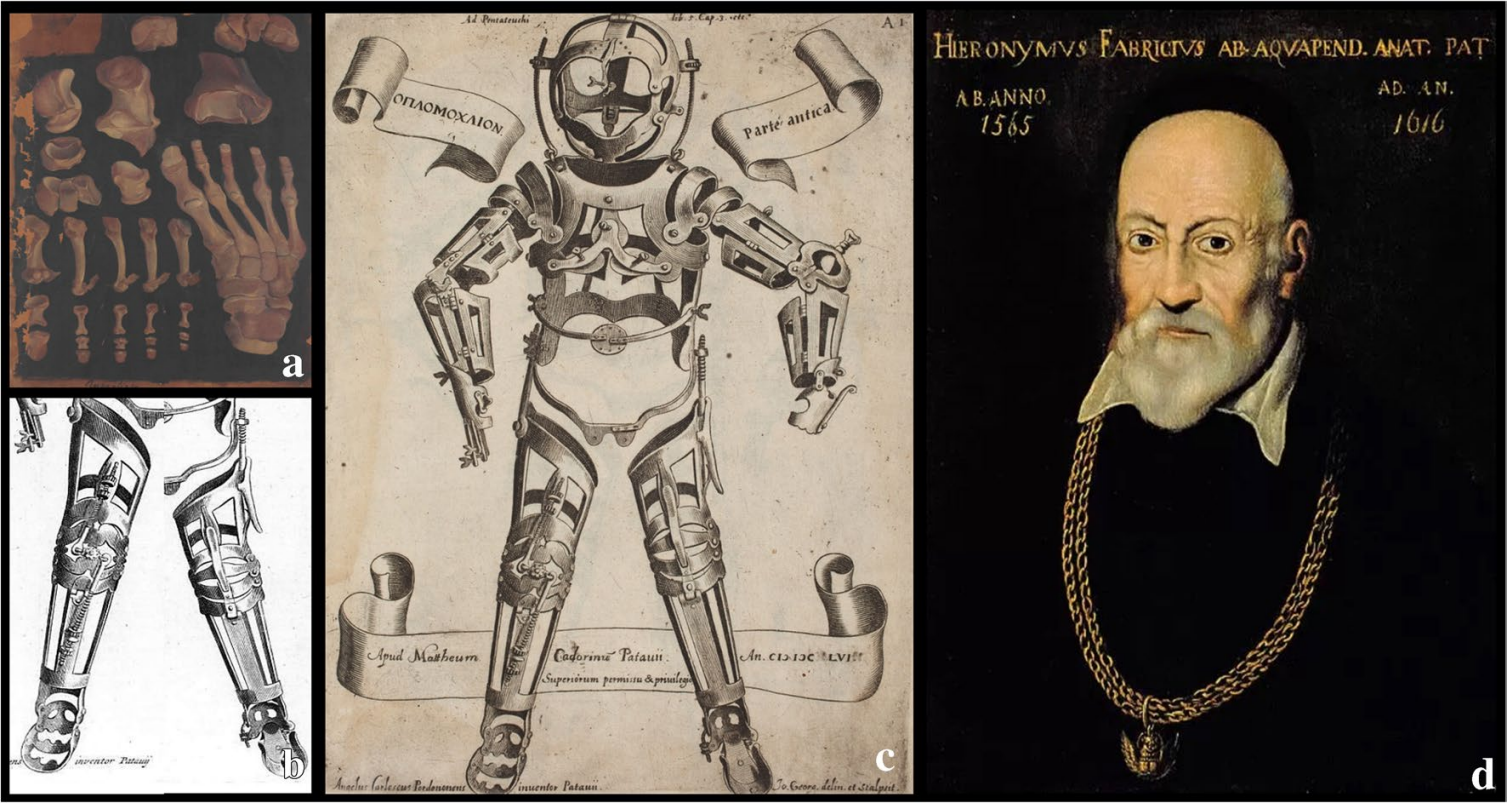
**a** Italian representation of right foot from dorsal view, 1575–1625, paint by G. F. d’Acquapendente, from “*Theatrum totius animalis fabricae – De anatomia ossium*”*. Rari 111.21; Marciana National Library in Venice*; **b** inferior part (legs) and **c** complete anterior view (pars antica) of the Oplomochlion form Fabrici’s *Operationes chirurgicae* (1647); **d** anonymous, Portrait of Girolamo Fabrici d'Acquapendente *(Insignia of the Order of St. Mark of the Republic of Venice)*

The Oplomochlion consisted of a collection of very diverse orthotic, prosthetic, and surgical metal instruments arranged with a demonstrative purpose and a topographic criterion. In its distal part, it has an apparatus for correcting various deformities of the feet, in particular valgus deformity of the hindfoot and congenital talipes equinovarus. He understood that the bone tissue, together with the tendons and ligaments, is only pliant and malleable in children and adolescents. For this reason, Acquapendente stated that malformations such as those mentioned previously and pes planus could only be corrected in very young individuals by using specific parts of his Oplomochlion [39] (Fig. 6).

## Conservative treatments: from antiquity to the twenty-first century

From ancient Egypt (3900 B.C.–342 A.D.) to the medieval period (500 A.D.–1400 A.D.), wood orthosis was the only treatment for *Hugh Owen Thomas *(Anglesey 1834–Liverpool 1891) and the management of pes planus. The first orthosis discovered throughout history is reported to come from the 5th Egyptian Dynasty, which is equivalent to 2750 B.C.–2625 B.C., and is a wood splint made for a fractured limb [40].

In the following periods, nothing can be found about the exact development of orthoses for flat feet in the literature. What we know is that materials have evolved and were later replaced with metal and leather which are either heavy, bulky, or thick, making them uncomfortable for the wearers. After the renaissance era (1400–1600) and the age of revolution (1700 –1850), the development of orthoses and prostheses showed a rapid change in shapes, structure, and composition [40].

**Fig. 7:**
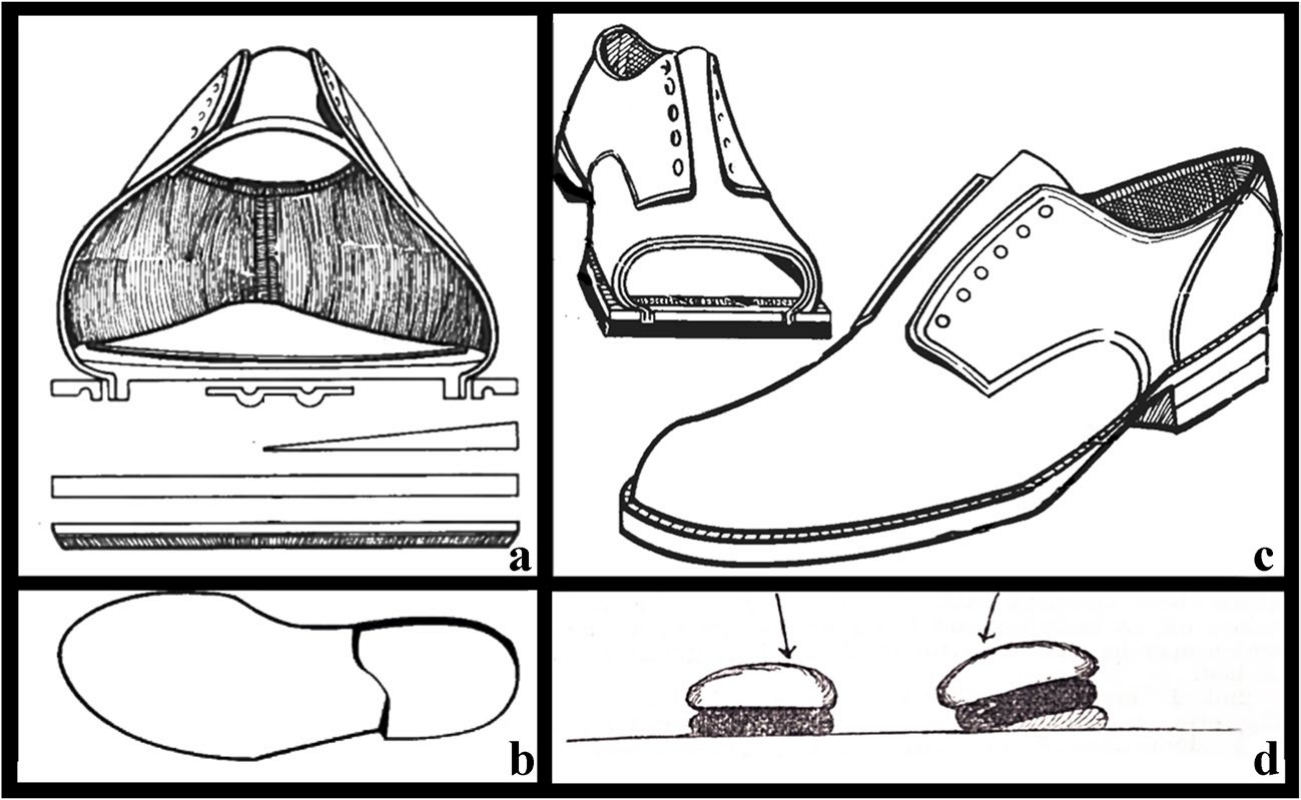
Early conservative treatments (nineteenth century): **a** anterior section of the Thomas heel; **b** isolated Thomas heel;** c** complete shoe with the Thomas heel; **d)** stylized representation of an early orthesis used for the varus/valgus correction

In 1876, Thomas invented a splint, which was used to treat deformities of the lower limb, including flatfoot. It was simple in design with the main objective of immobilising the lower limb. The splint was made from a padded metal ring attached to leather that was attached to an angled bar extending from the groin to below the foot on both sides of the leg. Basically, this device was a heel with an antero-medial extension [41] (longer than the standard) to add support to the medial arch of the midfoot [42] (Fig. 7).

In 1884, *Alexander Ogston *(Aberdeen 1844–Aberdeen 1929), an English surgeon remembered for being the first to describe *Staphylococcus aureus*, published an article in *The Bristol Medico-Chirurgical Journal* titled “On flatfoot, and its cure by operation” [43]. He wrote: “it is true that by rest and time the pain that accompanies the deformity becomes ameliorated or disappears, […] but the deformity does not disappear or even become materially diminished”. The therapy was therefore based on prolonged rest, with or without rigid bandages, and on the use of orthoses and braces. In particular, what was used was: “Boots with the inner margins of the soles raised, arched steel supports under the inner side of the sole, well moulded pads of cork and other materials, or hollow cushions of caoutchouc […]. Lateral supports to counteract the valgus position […]”. Another option was the so-called Langenbeck method, for child flatfoot: forced manual reduction of plantar flatness and subsequent immobilisation of the foot in plaster bandages for about three months [43–45].

Improvement in orthosis materials for flatfoot management occurred only after 100 years. During this period, the second industrial revolution (1870–1914) led to the discovery of plastic. In the 1960s, *Yates and Lehneis* wrote the first article about replacing metal orthoses with thermoformed plastics. It was a matter of discussion at first; however, after some investigation, it was found that plastic has more advantages than metal. It is light, hygienic, form-fitting, and noise-free. Unlike metal orthoses, plastic orthoses are thin enough to be worn under the user’s clothes, thus increasing the cosmetic value. Since then, thermoformed plastics have started to dominate the orthotic and prosthetic fields [46].

## First surgical proposals for flatfoot correction

**Fig. 8:**
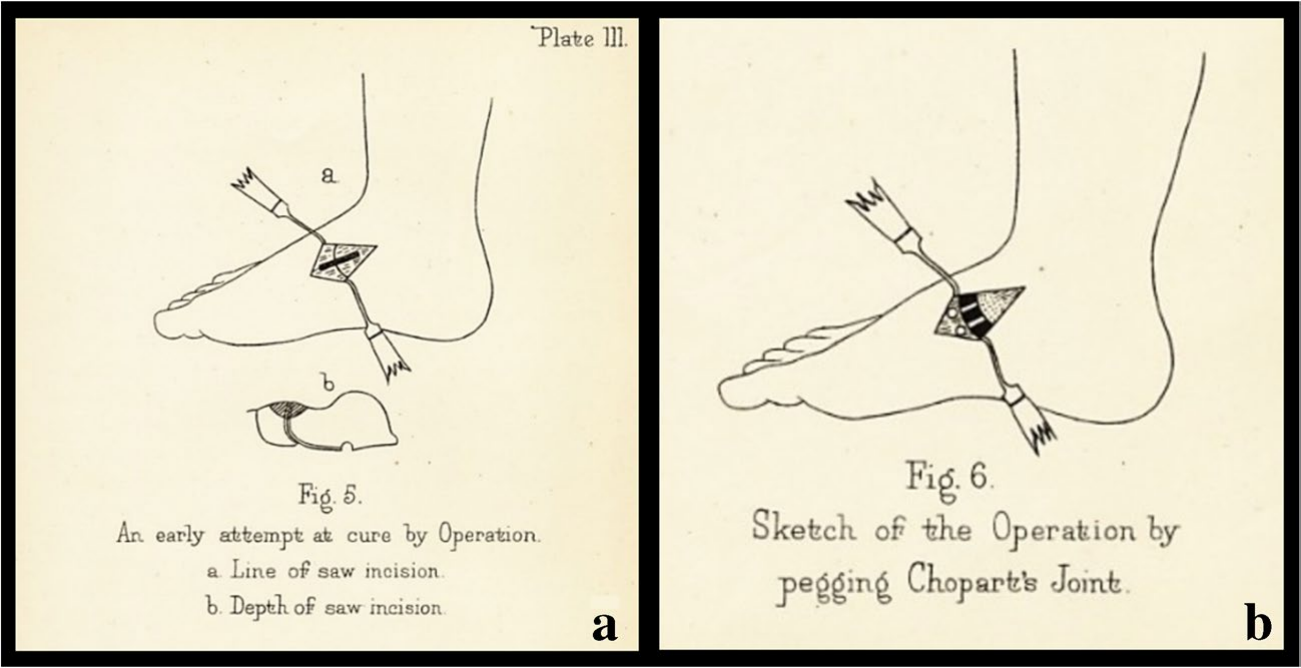
Surgical approach described by *Ogston in Bristol Med Chir J *(1884): **a** skin incision;** b** Chopart’s joint

The surgical technique, described by Ogston in 1884, for flatfoot correction consisted of a medial incision below the ankle, joint exposure, removal of articular parts of the scaphoid and talus, reduction of the deformity, and subsequent stabilisation with an ivory block placed between the two bones. In the post-operative period, plaster casts were applied and then removed after four days to four weeks with discharge (bed rest) for two to three months. The results were reported as excellent, as the deformity remained reduced over time [43] (Fig. 8).

In 1885, *William Stokes *(Dublin 1804–Dublin 1878), in his article “Astragaloid Osteotomy in the Treatment of Flat-Foot”, advised practicing the same incision without opening the ankle joint, modifying Ogston’s technique. He recommended performing an osteotomy to remove a wedge-shaped part of the head of the talus with an inferior base to restore the foot arch by abduction and supination. He expressed his scepticism about the flatfoot conservative treatment writing: “noticing how uniformly unsuccessful the attempts are to permanently remedy the deformity by any of the routine lines of practice, pushing up the arch of the foot and keeping it in that position by any of the various mechanical adjustments designed for this purpose” [44].

After this period, nothing can be found in the scientific literature over the subsequent 50 years regarding new deformity correction techniques until the second World War. In 1940, A.S. *Blundell Bankart *(Exter 1879–Northwood 1951) published a paper in *The British Medical Journal* titled “The treatment of flatfoot” in which he described six different types of flatfeet: the “congenital flatfoot”, presented at birth; the “Mobile flatfoot”, a deformity due to an incorrect posture typical of children and adolescents; the “Stiff flatfoot”, caused by fibrous adhesions, by tarsal coalition (fusion of two or more of the tarsal bones) [47], or by an increase in ligamentous thickness due to pre-existing reducible flatfoot or to outcomes of trauma or rheumatism; the “Contracted flatfoot”, fibrous flatfoot associated with claw toes and hallux valgus; the “Rigid (osseous) flatfoot”, caused by conditions of generalised arthrosis or outcomes of foot trauma; and finally the “Spasmodic flatfoot”, a typical condition of male adolescents between 13 and 18 years characterised by abduction and eversion of the foot, of unknown aetiology [48].

In the same article, Bankart gave useful indications for child flatfoot: “the treatment […] is uncertain and often disappointing. The most common method is manipulation under anaesthesia and fixation of the foot in plaster in the inverted position. […] Afterwards, he should wear an outside steel with a valgus T-strap to prevent passive eversion of the foot. In some very resistant cases, arthrodesis of the astragalo-scaphoid joint has been done” [48].

About 40 years later, in 1979, *Douglas C. S. Brown* wrote in the article titled “The Pediatric Foot”: “since many infant feet improve with arch supports, and many with flat feet do not have problems, it is hard to justify treating them. […] Certainly, the little scaphoid pads and 4 mm wedges do not harm, but combined with expensive shoes help perpetuate the myth that all this is necessary” [49].

## Modern and current operative procedures for flatfoot (20th–21st centuries)

It is now known that foot deformity differs in aetiology and treatment between children and adults. In most cases and when the patient is asymptomatic, it does not need therapy even if it is a severe form [50]. On the contrary, in symptomatic cases, most of the authors agree on surgical therapy because of the low efficacy of conservative treatment (corrective shoes, custom-made arch supports, custom-made insoles, among others) [51–53].

Until now, the most popular corrective procedures described have been osteotomies, arthrodesis of one or more foot and ankle joints, arthrorisis, tendon lengthening and transfers, and hindfoot correction surgery. Combinations of these procedures have also been reported.

### Osteotomies

Over time, the most widespread osteotomies and their variants have been numerous: the medial heel bone osteotomy; elongation of the lateral column through the calcaneus or cuboid; and osteotomy of the medial cuneiform, of the first metatarsal, and of the calcaneus.

Among the first described, *Trendelenburg *(1889) proposed a supramalleolar varising osteotomy of the fibula and tibia; *Garrè and Kuttner *(1914) recommend a personalised wedge resection of the talo-navicular joint; *Perthes *(1985) instead devised the “shaping double osteotomy”, an osteotomy with medial subtraction and lateral addition; *Wilms *(1919) recommended talonavicular arthrodesis and insertion of a bone wedge (taken from the head of the talus) into the calcaneocuboid joint.

The following are among the modern ones: *Chambres *(1946),* Baker and Hill *(1964), and *Selakovich *(1973) proposed additional subthalamic osteotomies; *Baker and Hill* recommended surgery for spastic neurologic flatfoot (1964); *Regnauld *(1974) performed an extra-articular osteotomy of the neck of the talus and the body of the scaphoid, lengthening the Achilles tendon [54]; the *Evans calcaneal osteotomy*, first described by *Dillwyn Evans *(Cardiff 1910–Cardiff 1974) in a post-mortem article published in 1975, resumed Perthes’ osteotomy, routinely used by foot and ankle surgeons to correct both paediatric and adult pes planovalgus deformities by performing an osteotomy in the neck of the calcaneus where a trapezoidal wedge of tricortical bone is placed [55].

Calcaneal osteotomy has become a reliable and widely used technique in the operative correction of hindfoot deformity [56]. The lateral approach originally described by *Atkins* in 1992 [57] has now been developed through anatomical cadaveric and clinical studies to improve wound healing and minimise the disruption to neighbouring neurovascular structures (sural nerve and branches of the peroneal artery). To reduce the rate of complications, a minimally invasive surgical (MIS) technique based on a low-speed, high-torque burr has been adopted in the last 20 years [56, 58, 59].

### Arthrodesis

The origins of the triple arthrodesis date back to the early 1900s when the procedure was aimed to treat different conditions: idiopathic pes cavus and planus, deformities related to paralytic conditions of the foot [60]. In 1908, *Royal Whitman *(Portland 1857–New York 1946) devised a surgical treatment to address calcaneovalgus deformity due to neuromuscular abnormalities. He described the removal of the talus and backward displacement of the foot, though it was not successful for calcaneovalgus deformity [61]. In 1921, *Hoke *(Lincolnton 1874–USA 1944) proposed a triple arthrodesis where a portion of the talus was resected and the residual part remodelled with the fusion of the subtalar and calcaneocuboid joints [62]. This technique was in use for about 50 years. In 1978, *J. W. Duncan* published an article in which he found a 6.5% rate of osteonecrosis of the talus when the talar head resection was performed proximal to the origin of the artery of the tarsal canal [63]. Hoke’s technique was successfully modified by performing the resection distal to the artery. This technique is no longer used as it has been seen to lead to degenerative changes in the nearby joints over time [64].

### Arthrorisis

The arthrorisis techniques involve the placement of an implant or bone graft within the tarsal sinus to limit subtalar joint movement, to improve the longitudinal arch, and to reduce the valgus of the subtalar joint. Arthrorisis procedures were originally designed for paediatric treatment and generally involve joint-sparing techniques that correct the flatfoot deformity while preserving foot function. This approach stems from extraarticular subtalar arthrodesis, first described by *Grice* in 1952, grafting a block of corticocancellous bone harvested from the tibia or iliac crest [65]. Also *Chambers*, in 1946, proposed to fill the sinus tarsi with an autologous bone graft [66]. In 1962, *Haraldsson* wrote that the goal of the flatfoot correction procedure had not to be the block but the limitation of joint movement, a concept subsequently reaffirmed by *LeLièvre* in 1970 [67].

Carried out for the first time by Recaredo Alvarez [68, 69] in 1972, the calcaneostop technique was used by Burutaran [70] in 1979, he described the results achieved with this surgical method stabilizing the heel with astragalic rise, hindering the valgisation. Later, Pisani [71], by introducing some modifications on the positioning of the screw, obtained a double effect of remodulation of the foot with respect of the articular and sinus-tarsal structure, historically paving the way for new additional corrective solutions.

Four years later, *Subotnick* described the use of a silicone implant inserted into the sinus tarsi without being fixed [72], while in 1979, *Lanham* used the stem of a Swanson prosthesis. Later, *Volger* (1980) proposed to create housing in the calcaneus with a burr for the stem of the same implant to prevent its mobilisation. In 1983, *Smith and Millar* described the use of a polyethylene device (“STA-peg”) fixed in the heel [73, 74]. A few years later, *Giannini *et al. developed an expansive device to be inserted into the sinus tarsi, which they subsequently improved through the use of bioresorbable materials [75]. The *Maxwell–Brancheau* arthrorisis (MBA) implant, a large cylinder-shaped titanium screw, and the *Giannini* flatfoot expanding implant, a Teflon/stainless steel expansion drywall anchor design, are perhaps the most commonly used implants [75].

### Tendon lengthening and transfer

In 1936, *Kulowski* reported an effusion into the tendon sheath of the tibialis posterior tendon for the first time [76]; then in 1950, *Lapidus* and *Seidenstein* reported two cases of tenosynovitis of the same structure [77]. Subsequently, *Kettelkamp and Alexander* published the results of surgical exploration in adult patients with a typical combination of painful flatfoot deformity with tenderness and swelling along the sheath of the tibialis posterior tendon. They found in two of them that the tendon had ruptured in the mid-portion, and in one of them, it had avulsed from the navicular insertion [78]. In 1974, *Goldner* was one of the first surgeons to perform a replacement of the tibialis posterior tendon using flexor digitorum longus and flexor hallucis longus [79]. This is a case series on 9 patients, treated using the advancement of the posterior tibial tendon and plication of the medial ligaments to decrease the flatfoot deformity. Among these patients, three were teenagers (11, 15, and 18 years old) with traumatic flatfoot. All the others were middle aged with chronic tenosynovitis [79].

### Hindfoot correction surgery

The first technique of hindfoot correction surgery was described by *Golding-Bird* in 1888 and was based on scaphoid and/or astragalus head excision [80]. In 1927, *Miller* proposed a scaphoid-cuneiform and cuneiform-metatarsal arthrodesis with medial access to correct the deformity [81]. This procedure was modified by *Hoke*, introducing the first cuneiform osteotomy [82]. Meanwhile, *Durham* proposed a scapho-cuneiform arthrodesis stabilised with a K-wire or a screw [83]. In 1929, *Kidner and Albanese* reinserted plantarly and laterally the posterior tibial tendon with a medial resection of the scaphoid and the first cuneiform. Subsequently, *Natiello* came up with the tenodesis of the tibialis anterior and posterior, and *Pisani* described the removal of the calcaneal-scaphoid ligament tissue with a fixation of the tibialis posterior tendon.

## Conclusions

During the centuries, some therapeutic strategies, especially conservative ones, have never radically changed in their substance, while the operative ones have become the protagonists of an extraordinary evolution during the twentieth century up to the present. Nevertheless, after more than 2000 years of history, there is no consensus regarding the best solution for the flatfoot. This lack of a gold standard treatment seems to be the result of multiple points of view about pes planus, and many authors, also quoted in the present study, seem to have spent more time trying to identify the best corrective method rather than to clearly answer a crucial question on this issue: is flatfoot a pathology, anatomical condition, or a mere phenotypic feature of the human body?

## Data Availability

Any research materials of this study are available at our institution and can be accessed.
